# A Review of Placenta and Umbilical Cord-Derived Stem Cells and the Immunomodulatory Basis of Their Therapeutic Potential in Bronchopulmonary Dysplasia

**DOI:** 10.3389/fped.2021.615508

**Published:** 2021-03-09

**Authors:** Wai Kit Chia, Fook Choe Cheah, Nor Haslinda Abdul Aziz, Nirmala Chandralega Kampan, Salwati Shuib, Teck Yee Khong, Geok Chin Tan, Yin Ping Wong

**Affiliations:** ^1^Department of Pathology, Faculty of Medicine, Universiti Kebangsaan Malaysia, Kuala Lumpur, Malaysia; ^2^Department of Pediatrics, Faculty of Medicine, Universiti Kebangsaan Malaysia, Kuala Lumpur, Malaysia; ^3^Department of Obstetrics and Gynecology, Faculty of Medicine, Universiti Kebangsaan Malaysia, Kuala Lumpur, Malaysia; ^4^Department of Pathology, SA Pathology, Women's and Children's Hospital, Adelaide, SA, Australia

**Keywords:** bronchopulmonary dysplasia, placenta, regenerative medicine, stem cell, umbilical cord

## Abstract

Bronchopulmonary dysplasia (BPD) is a devastating lung disorder of preterm infants as a result of an aberrant reparative response following exposures to various antenatal and postnatal insults. Despite sophisticated medical treatment in this modern era, the incidence of BPD remains unabated. The current strategies to prevent and treat BPD have met with limited success. The emergence of stem cell therapy may be a potential breakthrough in mitigating this complex chronic lung disorder. Over the last two decades, the human placenta and umbilical cord have gained increasing attention as a highly potential source of stem cells. Placenta-derived stem cells (PDSCs) and umbilical cord-derived stem cells (UCDSCs) display several advantages such as immune tolerance and are generally devoid of ethical constraints, in addition to their stemness qualities. They possess the characteristics of both embryonic and mesenchymal stromal/stem cells. Recently, there are many preclinical studies investigating the use of these cells as therapeutic agents in neonatal disease models for clinical applications. In this review, we describe the preclinical and clinical studies using PDSCs and UCDSCs as treatment in animal models of BPD. The source of these stem cells, routes of administration, and effects on immunomodulation, inflammation and regeneration in the injured lung are also discussed. Lastly, a brief description summarized the completed and ongoing clinical trials using PDSCs and UCDSCs as therapeutic agents in preventing or treating BPD. Due to the complexity of BPD, the development of a safe and efficient therapeutic agent remains a major challenge to both clinicians and researchers.

## Introduction to Stem Cell Biology

Stem cell therapy has evolved tremendously since its first success story of bone marrow cells in regenerating a rodent's infarcted myocardium (1). In recent years, it has been advocated as a novel yet promising treatment modality for a myriad of diseases, including cardiovascular, neurodegenerative, musculoskeletal, wound repair. Stem cells are unspecialized cells in the human body that have a remarkable capability to regenerate continually. The key abilities of stem cells to constantly self-renew, proliferate and differentiate into specialized cells under adapted physiological environment allow them to restore tissue to its pre-injurious state (2). Sources of stem cells include bone marrow, umbilical cord, cord blood and adipose tissue.

Stem cell potency is defined as its capability to self-renew and differentiate, thus classified as totipotent, pluripotent, multipotent, and unipotent. The term plasticity means the ability to be molded or change to adapt to the situation. Stem cell plasticity is defined as the ability to give rise to different cell types (3, 4). The potency of stem cells reduces with each journey of lineage differentiation from early embryogenesis to mature specialized cells (4). A zygote, which is formed following ovum fertilization by a sperm, is the classic example of a totipotent stem cell. It has the ability to generate the embryonic as well as extra-embryonic structures including placenta. The blastocyst, which is formed 5 days after fertilization, consists of the inner cell mass (also known as embryoblast) rimmed by trophoblasts (5). The latter will develop into the placenta. Human embryonic stem cells (hESCs) which originate from the inner cell mass, remain undifferentiated with pluripotent potential. Similar to totipotent stem cells, pluripotent stem cells are able to give rise to all cell types of any of the three primary germ layers (endoderm, mesoderm and ectoderm) in the body, but lacking the capability to produce extra-embryonic cells. Following differentiation of pluripotent stem cells into one of the germ layers, they become multipotent stem cells with differentiation potential restricted to only cells of that germ layer (5). Adult and fetal stem cells are the examples of multipotent stem cells. Adult stem cells, or somatic stem cells are found in adult somatic tissues (6), whereas fetal stem cells are obtained from cadaveric fetuses following medically terminated pregnancies. Unipotent stem cell has the narrowest differentiation ability to only one cell type (5). Progenitor cells are descendants of stem cells with limited ability to differentiate and replicate. Hematopoietic, neural and cardiac progenitor cells are among the examples (7).

Several categories of stem cells have been widely investigated over the last few decades, such as embryonic stem cells (ESCs), fetal stem cells and somatic/adult stem cells. Acquisition of pluripotent ESCs that involved destruction of a developing embryos and the use of fetal stem cells from aborted/living fetal tissue however, posed some ethical and legal implications (8). Moreover, these pluripotent ESCs and possibly fetal stem cells which possess similar oncogenic properties with cancer stem cells raise a major safety concern as these cells may undergo undesirable differentiation and pose a risk of malignant transformation post transplantation (9). For instance, *in vivo* teratoma when grafted in severe combined immunodeficient mice (10).

Human placenta, an ephemeral but crucial organ in pregnancy, is an alternative reservoir of stem cells. Apart from its fundamental role in determining optimal fetal growth trajectory *in utero*, it represents a rich source of stem cells that could offer additional advantages in terms of proliferation and plasticity compared to adult stem cells (11, 12). Placenta and umbilical cord are traditionally regarded as nothing but biological waste, is often discarded after parturition. This helps to resolve the ethical concern inherent in ESCs (8, 13). Unlike stem cells harvested from other sources such as the bone marrow, adipose tissue and endometrium, these placental and umbilical cord tissues are readily available in large quantities and its stem-cell derivatives are easily recovered without the donors incurring any invasive surgical procedures (14). These unique features of PDSCs and UCDSCs make them attractive alternatives in cell therapies and regenerative medicine.

In this review, we first provide an overview of bronchopulmonary dysplasia (BPD) and the disease pathogenesis, followed by the potential roles of human PDSCs and UCDSCs as effective therapeutic and possibly preventive modalities for BPD as the disease in focus. Unanswered fundamental challenges related to clinical translation of stem cells from bench to bedside in BPD are also discussed.

## Bronchopulmonary Dysplasia and the Disease Pathogenesis

Infants born extremely premature may have arrested lung development at the canalicular-saccular phase, before alveolarization could occur. These infants are inevitably exposed to postnatal interventions such as positive pressure mechanical ventilation, supplemental oxygen therapy and sustaining recurrent bouts of infections that may exert further harmful effects on the immature lung. Consequently, the prematurity-induced interruption of normal alveolar and distal vascular development, which is superimposed by pulmonary inflammation and aberrant reparative process may collectively contribute to the progression of BPD (15).

The lung of infants with BPD has three histopathological features: [1] widespread but occasionally patchy interstitial edema and fibrosis may give rise to areas of relative collapse and fibrosis accompanied by more distended emphysematous lung and thus less alveolar surface area; [2] arterial muscular hypertrophy and adventitial thickening of small pulmonary arteries leading to increased vascular resistance and pulmonary hypertension; and [3] less usually nowadays, acute major airway pathology characterized by necrosis associated with an obliterative bronchiolitis, squamous metaplasia, and collapse of lung tissue distal to the obstructed airway (16).

### Imbalance Between Pro- and Anti-inflammatory Activities

An imbalance between pro- and anti-inflammatory activities in the lung is proposed as the major mechanism resulting in BPD (17). It is characterized by influx of neutrophils and macrophages into the airways and lung parenchyma. Migration and accumulation of these inflammatory components in the lung may amplify and perpetuate further immune activation by release of potentially destructive pro-inflammatory cytokines, chemokines and tissue proteases such as tumor necrosis factor-alpha (TNF-α), interleukin-8 (IL-8), IL-1, IL-6, matrix metalloproteinase-8 (MMP-8) and MMP-9 (17).

Besides, adhesion molecules such as soluble E-selectin (sE-selectin) and intercellular adhesion molecule-1 (ICAM-1) were found elevated in the arterial plasma level of infants with BPD (18, 19). These adhesion molecules initiate effective immune response by triggering leukocyte rolling, adhesion and transendothelial migration to the site of injury (20). This results in an influx of neutrophils to the lung tissue, followed by recruitment of macrophages, which in turn produce macrophage inflammatory proteins (MIP-1a and MIP-1b), causing a disturbance in pro- and anti-inflammatory factors in the lung (21).

The release of pro-inflammatory cytokines such as TNF-α, IL-1β, IL-6 and IL-8 activates NLR Family Pyrin Domain Containing 3 (NLRP3) inflammasome protein complex (22), incites the upregulation of nuclear factor kappa B (NF-κB) signal transduction pathway. Bourbia et al. (23) revealed a high NF-κB transcription factor concentration in the tracheobronchial lavage of infants with BPD compared to those without (23). Iosef et al. (24) demonstrated that NF-κB inhibitor engendered alveolar simplification with marked reduction in pulmonary capillary density of neonatal mice, similar to that seen in BPD. Taken together, these suggest the physiological role of NK-κB in the developing lung by promoting angiogenesis and alveolarization (24). Intriguingly, whether NF-κB possesses pro- or anti-inflammatory property depends on the timing and degree of stimuli as well as the maturation status of the lung. By inhibiting NF-κB after the onset of inflammation aggravates the inflammatory response, while inhibiting NF-κB prior to injury showed otherwise (25). Similarly, NF-κB inhibition in the neonatal lung increased the inflammatory response, while the same treatment in adult mice gave a protective effect by repressing inflammation (26).

### Aberrant Tissue Repair and Fibrosis

Inflammatory response is a double-edged sword, in which inflammation can be beneficial but at the same time detrimental to the host. Despite playing an active defense role against diverse insults and removing offending pathogens, exuberant inflammation can lead to paradoxical tissue destruction that trigger tissue repair, leading to fibrosis and scarring.

Developmental pathways particularly transforming growth factor-β (TGF-β) and Wnt signaling pathways that are involved in regulating various stages of lung development have been implicated in the pathogenesis of BPD. Wnt signal transduction cascade governs a myriad of developmental processes in the mammalian embryonic state, maintaining tissue homeostasis and controlling stem cells in postnatal life (27); while TGF-β is the key player in tissue healing by mediating fibroblastic activation, myofibroblastic transdifferentiation and extracellular matrix deposition.

### Abnormal Pulmonary Vasculogenesis

In addition to defects in airway remodeling, dysmorphic vascular growth pattern within the distal airways were observed in various animal models of BPD, while some pulmonary arteries underwent structural remodeling with medial hypertrophy and distal arterial muscularization which contribute to pulmonary hypertension (28). Several angiogenic growth factors, such as vascular endothelial growth factor (VEGF) and nitric oxide (NO) were noticeably reduced in their expression in experimental models of BPD.

### Dysfunction of Endogenous Lung-Resident Stem/Progenitor Cells

The discovery of resident undifferentiated stem/progenitor cell population residing in all organ systems including the lung, has opened up a new door to remarkable research in the field of regenerative medicine in recent years, with new knowledge on their physiological roles in lung development, pathogenesis and repair (29, 30) It is hypothesized that functional impairment or depletion of these lung-resident stem/progenitor cell populations has contributed to the disease pathogenesis in BPD (31).

Lung-resident stem/progenitor cells include cells of endothelial, mesenchymal and epithelial lineages. Endothelial progenitor cells are involved in vascular repair and regeneration by homing to the injury site to restore endothelial integrity and secure tissue perfusion. Endothelial colony-forming cells (ECFCs), a subset of endothelial progenitor cells with intrinsic self-renewal potential and capable of forming *de novo* vessels *in vivo* (32), were found to be in lower amount in the cord blood of infants with BPD (33). In contrast, those with high levels of ECFCs were protected from developing BPD, indicating its pivotal role in lung vascular maturation process. Baker et al. (34) revealed that ECFCs were highly susceptible to hyperoxia *in vitro* and lost their angiogenic potential. This observation linked the dysfunctional endogenous stem cell theory with occurrence of BPD in preterm neonates receiving postnatal oxygen therapy (34).

Lung-resident mesenchymal stromal/stem cells (L-MSCs) are stem cells found within the lung mesenchyme along with fibroblasts and extracellular matrix. They play a crucial role in the normal lung development and are recently discovered as the orchestrators in alveolarization through conduct of the tightly regulated processes of alveolar septation and angiogenesis in a developing lung (31, 35). In murine model, these L-MSCs were able to regenerate giving rise to differentiated daughter cells including airway smooth muscle cells or stalk mesenchyme fibroblasts (36). In addition, L-MSCs directly coordinate the proliferation and maturation of lung epithelial stem/progenitor cells via epithelial-mesenchymal crosstalk.

Lung epithelial stem/progenitor cells, like other stem cells, are capable of giving rise to differentiated cell lineages. For instance, alveolar type 2 epithelial cells exhibited the ability to proliferate and differentiate into alveolar type 1 epithelial cells in hyperoxia-induced rodent models (37), nonetheless, under the instruction of L-MSCs. Antenatal and repetitive postnatal insults cause damage and injury to these progenitor cells through various mechanisms. Failure of these cells to repair themselves in the desired manner leads to simplification of alveolar structures and abnormal pulmonary angiogenesis resulting in pulmonary hypertension.

It is strongly believed that targeting inflammatory-related and angiogenic signaling pathways could reduce the severity of BPD (38). Hence, translation research aimed at modulating inflammation and angiogenesis might be a new hope for an effective remedy to treat or prevent this devastating disease of premature newborns. The potential therapeutic value of cell-based replacement therapy acting as anti-inflammatory and excellent reparative agent to ameliorate BPD is further explored in the following section.

## Human Placenta and Umbilical Cord and Their Stem Cell Derivatives

### The Normal Human Placenta and Umbilical Cord

The eutherian mammalian placentas show striking morphological and structural diversity across species (39), and are classified as epitheliochorial, endotheliochorial or hemochorial according to the number of tissue layers separating the maternal blood from that of the fetus (40). Similar to rodents, rabbits and primates, humans possess a hemochorial placental subtype, characterized by negligible cellular barrier between the maternal and fetal circulations, thus allowing effective transfer of nutrients to fetuses (41). A normal term human placenta has a flat, round to elliptical, disc-like shape that measures ~22 and 2.5 cm in diameter and thickness, with an average weight of 500 g (42). It comprises both the fetus (chorionic plate) and maternal (basal plate) surfaces, held together by anchoring villi. These villi are organized into a series of 30 to 40 lobules or cotyledons, which are bathed directly by maternal blood filling the intervillous space, and epitomize the most important functional units for maternal-fetal exchange (43).

The outer surface of the chorionic villi constitutes the main cellular barrier between the fetal and maternal circulations and is formed by an outer layer of syncytiotrophoblasts and an inner layer of cytotrophoblasts, the latter of which diminishes as gestation progresses. The stroma of the villi is composed of sinusoidally dilated fetal capillaries embedded within loose connective tissues formed by mesenchymal cells, mesenchymal-derived macrophages (Hofbauer cells) and fibroblasts (44). The fetal membrane encompasses three distinct layers: [1] innermost amnion, [2] chorion laeve connective tissue, and [3] outermost decidua capsularis. Amnion comprises a single avascular layer of epithelial cells and connective tissue that contacts directly with the amniotic fluid and encloses the fetus. The chorion laeve is usually atrophic, composed of connective tissue containing fetal (chorio-allantoic) blood vessels, whereby decidua capsularis represents maternal modified endometrium (45). The developing embryo is connected to the chorionic plate (fetal surface) of placenta by an umbilical cord containing two arteries and one vein surrounded by gelatin-like mucoid substance, the Wharton's jelly. Wharton's jelly encompasses all loose connective tissue from the external surface of the tunica media of cord vessels to the inner margin of the amniotic epithelium (46). Wharton's jelly is important in keeping the integrity of umbilical cord. It prevents kinking of the cord and protects the umbilical blood vessels (47). The umbilical cord has an average length of 50 to 60 cm and diameter of 2 cm, with up to 40 helical turns (42).

### Placenta and Umbilical Cord-Derived Stem Cells

The human placenta and umbilical cord represent a reliably high yield reservoir of stem cells compared to other sources. The observation that teratoma can arise from a term placenta suggests that it may harbor some multipotent germ cells (48). Studies have reported the placenta contains a population of multipotent stem cells that express stem cells markers such as c-KIT, octamer-binding transcription factor 4 (OCT4), sex determining region Y-box 2 (SOX2), stage-specific embryonic antigen-3 (SSEA3), SSEA4, T cell receptor alpha locus-1-60 (TRA-1-60) and TRA-1-81 (11). These cells possess mesodermal phenotype and demonstrate broad multilineage differentiation ability (12, 49, 50).

**Figure 1:**
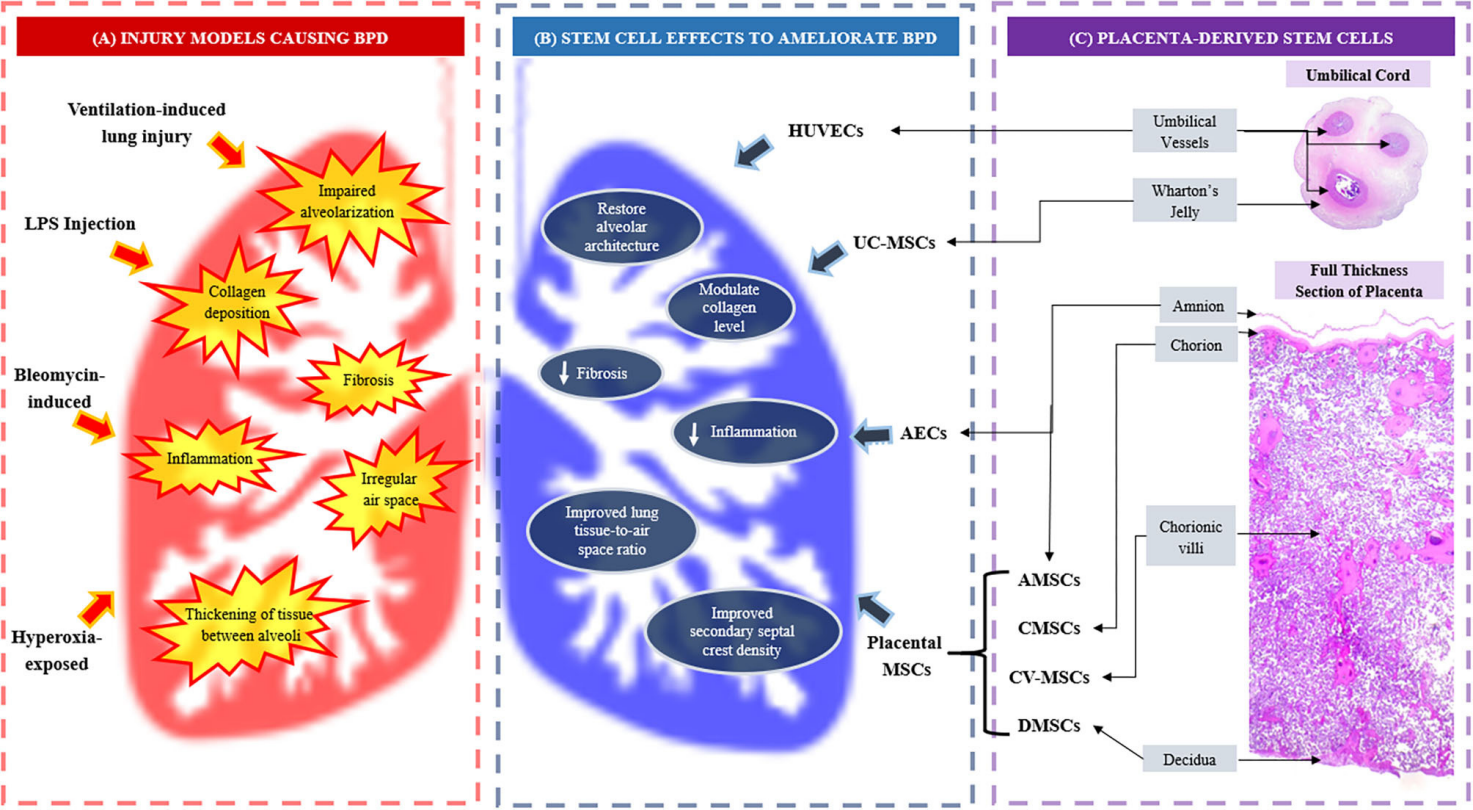
**(A)** Injury models causing bronchopulmonary dysplasia (BPD) and BPD-like injuries; **(B)** Effects of placenta and umbilical cord-derived stem cells in ameliorating BPD, and **(C)** The origin of placenta and umbilical cord-derived stem cells. AECs, amniotic epithelial cells; AMSCs, amniotic mesenchymal stromal/stem cells; BPD, bronchopulmonary dysplasia; CMSCs, chorionic mesenchymal stromal/stem cells; CV-MSCs, chorionic villi mesenchymal stromal/stem cells; DMSCs, decidual mesenchymal stromal/stem cells; LPS, lipopolysaccharide; UC-MSCs, umbilical cord mesenchymal stromal/stem cells.

Different sources of placental stem/progenitor cells are derived from different layers of the placenta, namely amnion, chorion and decidua (51) as well as umbilical cord which constitutes the Wharton's jelly and cord blood vessels (52) (Figure 1). The two types of stem cells from the amniotic layer are amniotic epithelial cells (AECs), which originate from the epiblast and amniotic mesenchymal stromal/stem cells (AMSCs), derived from the hypoblast (53, 54). Deriving from the chorion sheets are chorionic MSCs from the inner chorionic mesoderm, which is similar to mesenchymal region of the amnion, and chorionic trophoblast cells from the outer layer of trophoblastic origin. The uterine component of the placenta, the decidua, also harbors decidual MSCs (55). Wharton's jelly of the umbilical cord serves as attractive source of MSCs while cord lining membrane (amniotic epithelium and subamniotic Wharton's jelly) is identified as a valuable source of epithelial stem cells and MSCs, respectively (52). The PDSCs and UCDSCs may be utilized in a wide range of clinical applications. In this review, we focused on stem cells harvested from placenta and umbilical cord that have demonstrated potential therapeutic value in BPD.

#### Human Amniotic Epithelial Cells (hAECs)

The human amniotic epithelial cells (hAECs) are valuable progenitor/stem cells for regenerative medicine as these cells offer great promise for therapeutic application due to their ease of isolation, multilineage potential, immune privilege, anti-inflammatory properties, do not have telomerase reverse transcriptase, show a stable karyotype, do not form tumors when injected and have a low risk of allogeneic rejection (2, 53, 56).

Cell surface antigens and other specific markers are often used to define the “stemness” of a cell type. Investigations demonstrated that hAECs displayed a similar set of stem cell marker profile to that of ESCs. hAECs expressed most of the ESCs transcription factors and cell surface markers in the early second trimester, namely OCT4, NANOG, SOX2, SOX3, SSEA3, SSEA4, TRA*-*1-60, TRA-1-81, and GCTM2 (53, 56, 57). Although most of these cells with stem cell markers are lost over time, some of them are still retained in the term placental amniotic epithelium (58). hAECs are pluripotent and have unlimited capacity for self-renewal as well as the ability to differentiate into the derivatives of the three primary germ layers; ectoderm, mesoderm and endoderm (53, 56, 58, 59).

Studies showed that hAECs have low expression of the major histocompatibility complex (MHC) class I molecules [human leukocyte antigen (HLA)-A, HLA-B, HLA-C and β2 microglobulin] and MHC class II molecules (HLA-DR) (58, 60, 61). Since the amnion does not express MHC class II antigens, hAECs can elude the immune system (51). The other suggested mechanism of induction of tolerance of hAECs is related to the expression of unique HLA class 1b molecules (62). These HLA class 1b molecules regulate immune response under autoimmune and transplantation conditions, while HLA class I and II molecules contribute to the allogeneic immune rejection (58).

hAECs express a number of distinct Toll-like receptors (TLRs) and induce the production of inflammatory cytokines as a part of their immunomodulatory responses. TLR4 expression induces apoptosis, and play a role in the pathogenesis of premature rupture of membrane. Study showed TLR4 expression in hAECs was accompanied by reduced BCL-2 expression and increased Bax protein (63). TLR5 and TLR6 recognize a variety of pathogens. Both TLR2 and TLR6 are expressed in respond to Mycoplasma-associated protein, while TLR5 ligand is expressed in reaction with flagellated Gram-positive and Gram-negative bacteria. Activation of TLR5 and TLR6 induced proinflammatory response. Together, these indicate hAECs possess self-regulatory mechanisms in immunomodulatory responses (58).

#### Placental Mesenchymal Stromal/Stem Cells (P-MSCs)

Human MSCs were first described by Friedenstein in the bone marrow in 1968 (64). Although bone marrow remains the most common source of MSCs, these cells can also be isolated from various human tissues, such as the lung (30), adipose tissue (65), umbilical cord (66), skeletal muscle (67), dental pulp (68), corneal stroma (69), synovium (70), cardiac tissue (71), spleen, liver, kidney (72), bone marrow (73), cord blood (74), amniotic fluid (75), and placenta (76, 77). The placental mesenchymal stromal/stem cells (P-MSCs) express stromal markers, and are negative for the hematopoietic markers (78). Additionally, some studies reported P-MSCs expressed pluripotency markers (54, 79).

P-MSCs were first described in 2004 as plastic-adherent cells that share a similar immunophenotype with that of bone marrow MSCs and have multilineage differentiation potential (80). P-MSCs are of mesodermal origin. Depending on the layer they originate from, their stem cell derivatives include AMSCs, chorionic MSCs, chorionic villi MSCs and decidual MSCs (78, 81, 82). AMSCs (11, 83), chorionic MSCs (82, 84) and chorionic villi MSCs (78, 85) have been described as having a longer life span than the decidual MSCs population obtained from the maternal-derived decidua (82, 84).

P-MSCs have a much-limited differentiation repertoire compared to the pluripotent ESCs. These cells display the ability to differentiate *in vitro* into different mesodermal cell lineages, including adipocytes, chondrocytes, and osteoblasts (86). Another study suggested that P-MSCs may also be capable of neural differentiation (87). P-MSCs are widely studied in regenerative medicine. Other than their advantages in terms of the ease in isolation, high plasticity, low immunogenicity and tumorigenesis (51), P-MSCs display the ability to migrate to inflammatory microenvironments and tumors, involvement in angiogenesis and wound healing as well as tissue repair activity through paracrine actions, which are important therapeutic advantages of P-MSCs (88–90).

#### Umbilical Cord-Derived Stem Cells

Wharton's jelly is a popular source of umbilical cord mesenchymal stromal/stem cells (UC-MSCs) (91). Stem cells isolated from the Wharton's jelly show mesenchymal fibroblast-like morphology, with self-renewal ability and capable to differentiate into neuronal, osteo-chondral, adipocytic and muscular derivatives (92). Apart from having characteristics of MSCs as defined by the International Society for Cellular Therapy (93), UC-MSCs also exhibit properties attributed to ESCs, expressing markers such as TRA-1-60, TRA-1-81, SSEA1, SSEA-4 and alkaline phosphatase. In addition, ESC pluripotent markers that include OCT4, Sox-2, and NANOG are also detected at a lower level (94). Although UC-MSCs are not as pluripotent as ESCs, these cells are widely multipotent and do not develop into teratomas in immunocompromised mice (92).

Recently, Davies et al. (2017) proposed an anatomically/histologically-based nomenclature of the umbilical cord structures for the purpose of standardization and ease of comparison across cells harvested from different regions/zones of Wharton's jelly (46). Wharton's jelly is further divided into three distinct anatomical/histological zones: [1] subamnion, [2] intervascular and [3] perivascular Wharton's jelly. Interestingly, the stromal cells of Wharton's jelly are not uniformly distributed, but in a gradually increasing manner from the cord lining up to the proximity of umbilical vessels. Likewise, the tendency of myofibroblast differentiation of the stroma cells is the highest near the umbilical vessels with the least differentiated ones predominantly located in the subamniotic zone (95).

Accumulating evidence suggests that umbilical cord contains a unique cell family with different degree of stemness and phenotypic profiles residing in various parts of the umbilical cord (96). For instance, other than MSC markers (CD44, CD90 and CD105), stem cells harvested from perivascular Wharton's jelly demonstrated high expression of endothelial markers CD146 like that of CD146+ pericyte, a more differentiated MSC progenitor cells. They are however absent for CD73, a MSC marker. On the contrary, the intervascular Wharton's jelly yields stem cells positive for MSC markers but lacking that of endothelial markers (CD144, CD146 and CD34). The pericyte-like properties of the MSCs isolated from perivascular Wharton's jelly provide additional advantage in its rapid response to tissue damage upon engraftment and induce angiogenesis (97).

### Comparison Between Placenta/Umbilical Cord-Derived Mesenchymal Stromal/Stem Cells and Bone Marrow-Derived Mesenchymal Stromal/Stem Cells

#### In vitro Comparison

Stem cells harvested from bone marrow are considered as the gold standard and the most characterized source of adult MSCs for various clinical applications. Acquisition of MSCs from bone marrow; however, requires invasive procedure with a risk of complications. Moreover, the cell yield from bone marrow declines with advancing donor age. When compared to young donor (<40 years old), older donors' MSCs revealed smaller-sized colonies and lower integrated density (98).

The P-MSCs and UC-MSCs are excellent alternatives to BM-MSCs. The former is shown to share similar morphology, cell surface markers and some pluripotency-related markers with BM-MSCs. Interestingly, BD-MSCs have additional advantages than BM-MSCs. For instance, a higher frequency of colony forming unit-fibroblasts was reported in UC nucleated cells (1:1609) compared to BM nucleated cells (1:35700) (99). Lower immunogenicity was seen in UC-MSCs over BM-MSCs with lower levels of lymphocyte proliferation following allogeneic lymphocyte stimulation assay. Likewise, UC-MSCs have a higher overall immunomodulatory effect with increased expression of potent immunosuppressive factors such as CD200, LIF, and TGF-β2 (99). *In vitro* study showed UC-MSCs underwent slower senescence, demonstrated a higher cell proliferation rate and greater anti-inflammatory effects than BM-MSCs (100). The challenges remain in understanding the heterogeneity of the isolated populations, and to standardize the diversity in isolation protocols and culture conditions (101).

P-MSCs were observed to consistently faster population doubling time and longer-term expandability (up to 15 passages) under identical culture condition compared with BM-MSCs (102). P-MSCs are more homogeneous as compared to BM-MSCs in culture (103). This might be due to the fact that P-MSCs are younger cells with less exposure to harmful substances such as reactive oxygen species, chemical and biological agents, and physical stressors (104), thus increasing the efficacy and safety of the therapeutic applications of P-MSCs in regenerative medicine. Homing of P-MSCs to damaged tissue may be further enhanced with the demonstration of higher expression of VLA-4 (very late antigen-4) adhesion molecule on P-MSCs compared to BM-MSCs to allow adherence and migration through the endothelium (105). In addition, the advantage of P-MSCs over BM-MSCs and adipose tissue-derived stem cells includes the ability to be obtained using a non-invasive method and in larger quantity (106). Di Bernardo et al. (107) reported that P-MSCs played a pivotal role as potent stimulator of perinatal lung morphogenesis in *ex-vivo* fetal lung culture model compared with BM-MSCs (107).

#### *In vivo* Comparison

The therapeutic efficacy of BM-MSCs has been investigated in experimental models of lung injury in adult and newborn animals. Tian et al. (108) reported a significant improvement in radial alveolar count and reduction in lung inflammatory cytokines in hyperoxic mice model after intravenous BM-MSCs injection (108). Their findings were confirmed by other studies on hyperoxic rat models (109–111). Intra-tracheal delivery of BM-MSCs in hyperoxic neonatal rat lungs shown to confer protection with increased pulmonary vascular angiogenesis, reduced pulmonary hypertension and normalized alveolar structures (112). Conversely, intranasal administration of BM-MSCs in newborn mice lung injury model, failed to achieve epithelial reconstruction and transdifferentiation into respiratory epithelial cells (113). To date, the efficacy of BM-MSCs in larger animal BPD models such as preterm lambs, pigs and baboons have not been explored (114).

Although there have been extensive pre-clinical studies of BPD animal models using stem cells derived from bone marrow, placenta and umbilical cord, to the best of our knowledge, head-to-head comparative study on the efficacy of both the BM-MSCs and P-MSCs/UC-MSCs in BPD animal models is still lacking. This research gap needs to be further addressed.

## Mechanisms of Function of Placenta and Umbilical Cord-Derived Stem Cells

### Cell-Contact-Mediated Effects

Upon transplantation, MSCs exert their immunomodulatory functions at damaged sites through a synergy of direct cell-cell contact. The direct cell-to-cell contact between PD-1 inhibitory molecule on T cells and its ligands PD-L1 on MSCs, inhibits CD3+ T cell proliferation, induces early apoptosis and suppressed effector T cell (e.g., IL-17 producing T cells, Th17) responses (115). Similarly, TNF receptor superfamily member 6 (Fas)-FasL interactions propagate the death signal and induce T cell apoptosis (116). In addition, expression of CD106 (VCAM-1) on P-MSCs (117) and CD54 (ICAM-1) on UC-MSCs (118) is crucial in mediating immunomodulatory functions on T cells.

### Paracrine and Extracellular Vesicles-Mediated Effects

Despite the relatively poor *in vivo* engraftment rate (0–20%), pleiotropic lung protection following transplantation is believed to be attributed to paracrine factors such as lipid based mediators, growth factors and signaling peptides (119). Among the secreted bioactive substances include lipoxin A_4_ (120), epithelial growth factors (e.g., keratinocyte growth factor, pro-angiogenic factors) (121) and TNF-α-stimulated gene/protein 6 (122), which have potent anti-inflammatory properties. With the presence of these molecules, administered stem cells are able to migrate to injured tissue and promote anti-inflammatory environment which support cell proliferation and inhibit apoptosis, thus enhance tissue regeneration, remodeling and survival.

MSCs influence both tissue resident stem cells and macrophages through paracrine effects like extracellular vesicles (EVs) other than cytokines and secreted soluble factors, which results in more efficient reparative process by tissue resident stem cells (123). EVs (such as exosomes and microvesicles) are nanometer-scale, cell membrane-enclosed packages of biomolecules that are released by cells into the surrounding environment to mediate signal transmission and cell-to-cell communication. These vesicular cargo biomolecules carry biological active compounds including amino acids, bioactive lipids and nuclei acids (124). These cell-free products may serve as safer alternatives to cell therapies. For large scale production, adipose tissue-derived MSCs and UC-MSCs exosomes will be easier to obtain compare to BM-MSCs (123). Compared to BM-MSCs, UC-MSCs have a higher production rate of EVs (125).

Kourembanas team was the first to discover pleiotropic protective effects of EVs-based therapy in experimental BPD models. In their study, they reported that intravenous administration of EVs derived from both BM-MSCs and UC-MSCs in hypoxic mice model were able to inhibit influx of alveolar macrophages and pro-inflammatory cytokines, besides ameliorate lung vascular remodeling and pulmonary hypertension. Moreover, the uptakes by macrophages cause a shift in balance to anti-inflammatory state (126). Following that, the same group of researchers reported that the lung functions of the EVs-treated mice were significantly improved with decreased fibrosis, arteriole muscularization and pulmonary hypertension. They concluded that restoration of lung function occurred partly related to macrophage immunomodulation induced by administration of EVs (127). Several subsequent studies have shown that early administration of human MSC-derived EVs improved histological and functional outcomes in experimental BPD (122, 128).

Similar therapeutic effects were observed in hyperoxia-induced neonatal lung injury in mice treated both with early and late EVs interventions (129). EVs might even potentially reverse the cardiorespiratory complications in children with developed BPD (129). In addition, Tan et al. (130) reported that the release of EVs from hAECs administered intranasally exerted similar beneficial effects to MSCs with significant reduction in lung inflammation, improvement in tissue-to-airspace ratio and reduction in fibrosis in bleomycin-challenged aged mice (130). Willis et al. (129) and Bonadies et al. (131) suggested that MSCs and EVs are two most revolutionary treatments for BPD, not only effective in the prevention of BPD but also may potentially reverse the complications in children diagnosed with BPD (129, 131).

## Therapeutic Potential of Placenta and Umbilical Cord-Derived Stem Cells in Bronchopulmonary Dysplasia

### Experimental Therapeutics in Animal Models

Animal models play a crucial role in advancing our fundamental knowledge on the pathogenesis of this complex disease and to direct our future clinical trials into therapeutics. Mice and rats are among the widely used models since the newborn rodent pups are delivered at term in the saccular stage of lung development, equivalent to that of a preterm infant at risk of BPD. Other animal models for BPD that have been established are pigs (132), rabbits (133), lambs (134), and baboons (135). Arguably, larger animal models that require the need for mechanical ventilation and other life-support care after premature delivery are more clinically relevant and closely resemble human biology than rodents. Nonetheless, the use of these larger animal models increases the costs of experimentation and more labor intense, which might have precluded their use in preclinical studies (136). Methods of inducing BPD in animal models vary across studies and include the use of high concentration of oxygen (hyperoxia), mechanical ventilation, bleomycin, intrauterine inflammation via lipopolysaccharide (LPS) injection, postnatal continuous hypoxia or intrauterine prenatal hypoxia (137). Hyperoxia-induced lung injury is among the most used method.

Nonetheless, these animal models are not perfect representation of BPD in human. Induced animal models demonstrating alveolar simplification and vascular remodeling, may also reveal additional widespread fibrosis and inflammation (138). Recently, Zhang et al. (139) successfully created an innovative BPD model using premature hyperoxia-exposed rodents which showed characteristic histological features of BPD in humans (139), suggesting a new alternative model for future research. Table 1 summarizes the effects of PDSCs and UCDSCs in BPD therapeutic experimental animal models while Figure 1 illustrates how PDSCs/UCDSCs remedy the various BPD-like injuries inflicted in the models.

**Table 1:** Effects of placenta and umbilical cord-derived stem cells in BPD models.

**Cell types**	**Effects**	**Evidence**	**References**
hAECs	Anti-inflammatory	↓ Pro-inflammatory cytokines - TNF-α, TGF-β, IFN-γ, PDGF-α, PDGF-β, IL-1β, IL-10, IL-6 ↓ infiltration of inflammatory cells ↑ anti-inflammatory substances	(140–145)
	Anti-fibrosis	↓ collagen density	
	Lung function improvement	↓ peripheral pulmonary arterial remodeling ↑ density of pulmonary capillary bed Improved lung tissue-to-air space ratio Improved secondary septal crest density Restoration of alveolar architecture Preserved spatial pattern of elastin deposition Promoted pulmonary angiogenesis	
P-MSCs	Anti-inflammatory	↓ pro-inflammatory cytokines - IL-6, TNF-α	(146, 147)
	Anti-fibrosis	↑ VEGF ↓ CTGF ↓ collagen density ↓ infiltrating macrophages ↓ neutrophil infiltration	
	Lung function improvement	Restored vascular density	
hUC-MSCs	Anti-inflammatory	↓ Pro-inflammatory cytokines - TGF-β, IFN-γ, macrophage MIF and TNF-α	(148–151)
	Anti-fibrosis & ameliorate elastin remodeling	↓ Collagen density ↓ MMPs ↓ Elastin expression ↑ VEGF ↑ MMP-2 ↑ Vessel density ↑ Angiogenesis	
	Lung function improvement & accelerated repair	↓ BPD injury-related proteins - CX3CL1, TNF-α, TIM-1, hepassocin, neprilysin,osteoprotegerin, MMP-2, LIF ↑ alveolar septal widening ↑ septal crest density Restored lung alveolarization, vascularization and pulmonary vascular remodeling.	

*BPD, bronchopulmonary dysplasia; CTGF, connective tissue growth factor; CX3CL1, C-X3-C motif chemokine ligand 1; hAECs, human amniotic epithelial cells; hUC-MSCs, human umbilical cord mesenchymal stromal/stem cells; IFN-γ, interferon-γ; IL, interleukin; LIF, leukocyte inhibitory factor; MIF, migratory inhibitory factor; MMPs, matrix metalloproteinases; P-MSCs, placental mesenchymal stromal/stem cells; PDGF, platelet derived growth factors; SMA, smooth muscle actin; TGF-β, transforming growth factor-β; TIM-1, T cell immunoglobulin and mucin domain; TNF-α, tumor necrosis factor-α; VEGF, vascular endothelial growth factor*.

#### Human Amniotic Epithelial Cells (hAECs)

hAECs have been shown to reduce acute inflammation, accelerate repair and improve lung function in both immunodeficient and immunocompetent-mouse models with bleomycin-induced injury (140–142). Administration of hAECs to BPD models resulted in decreased gene expression of pro-inflammatory cytokines (TNF-α, TGF-β, IFN-γ, and IL-6), and decreased inflammatory cell infiltration (141). hAECs reduced scarring in lung injury by decreasing the collagen content (140). These cells were capable of mitigating lung inflammation and alveolar simplification in a murine model with BPD-like lung injury, by improving lung tissue-to-air space ratio and secondary septal crest density (143).

In larger animal like fetal sheep models, exposed to intraamniotic LPS injection, hAECs reduced the need of ventilation and reduced inflammatory changes (144, 145). In addition, it restored a normal lung tissue-to-air space ratio, reduced pro-inflammatory cytokines (145), normalized secondary septal crests, reduced collagen and elastin deposition and fibrosis (144). Deus et al. (152) described hAECs having beneficial effects by the production and secretion of various bioactive factors involved in anti-inflammation, immunomodulation, wound healing, angiogenesis, anti-fibrosis and anti-bacterial (152).

Studies showed host macrophages and T regulatory cells were the main contributors toward the reparative effects of hAECs (120, 153–155). Of note, the response to these cells was dependent on the timing of administration and the effects were best observed when they were administered at an early stage of injury (142, 143). Umezawa et al. (156) showed that placental amnion-derived cells can be reprogrammed to induced pluripotent stem cells. These cells maintained normal karyotype and chromosomal stability over a long period of passages. It can be easily isolated and expanded for industrial production of large quantities (156).

#### Placental Mesenchymal Stromal/Stem Cells (Placental MSCs)

Although not as widely utilized, P-MSCs attenuated perinatal inflammation- and hyperoxia-induced defective alveolarization and angiogenesis as well as reduced lung fibrosis. In LPS-injected rats, human MSCs derived from placentas improved vascular density, reduced TNF-α and IL-6 levels and collagen density, by exerting paracrine effects via increased VEGF and decreased connective tissue growth factor (CTGF) expression (146).

Cargnoni et al. (147) demonstrated that P-MSCs derived from fetal membrane showed stem cell phenotype, high plasticity, and displayed low immunogenicity both *in vitro* and *in vivo*. These MSCs also displayed the ability to engraft in the lung. A 1:1 mixture of hAECs and human AMSCs/human chorionic MSCs administered intratracheally into bleomycin-treated, immunocompetent C57/Bl6 mice exhibited a reduction in neutrophil infiltration and fibrosis, indicating the presence of the anti-fibrotic effect of PDSCs (147).

#### Umbilical Cord Mesenchymal Stromal/Stem Cells (UC-MSCs)

Umbilical cord mesenchymal stromal/stem cells (UC-MSCs) were injected into bleomycin-induced lung injury models and demonstrated reduced inflammation and fibrosis, with the injected cells found after 2 weeks and only in areas of inflammation and fibrosis (148). The treatment inhibited the expression of TGF-β, IFN-γ and proinflammatory cytokines macrophage migration inhibitory factor (MIF) and TNF-α. Collagen level was decreased, caused by up-regulation of MMP-2 and reduced endogenous inhibitors, tissue inhibitors of MMPs. These results suggest that UC-MSCs harbor anti inflammation and antifibrotic properties and may augment lung repair (148).

In hyperoxia-exposed newborn mice, intraperitoneal administration of UC-MSCs at high dose (1 x 10^6^ cells) restored lung structure and function (149). Characteristic arrest in alveolar growth with air space enlargement and loss of lung capillaries induced by hyperoxia were partially prevented and lung function and structure were somewhat preserved, without tumor formation following computed tomography scan assessment. While high dosage of intraperitoneal administration of UC-MSCs (1 × 10^6^ cells) was associated with alveolar septal widening probably through the modification of the interstitial matrix, intranasal administration of UC-MSCs or lower dose at 0.1 × 10^6^ cells intraperitoneal administration had no significant effects on lung function or alveolar remodeling. It is suggested that UC-MSCs may act via a paracrine effect. Purified exosomes from various sources of MSCs including Wharton's jelly-derived MSCs were also reported to restore lung architecture and improve lung development and function in hyperoxia-induced BPD animal models (129).

Moreira et al. (150) reported that the first intranasal administration of umbilical cord Wharton's jelly-derived MSC (UC-MSCs) to a hyperoxia-induced rat BPD model resulted in restoration of lung alveolarization, vascularization and pulmonary vascular remodeling. This was due to the combined effect on angiogenesis, immunomodulation, wound healing and cell survival of hUC-MSCs as indicated by the protein microarray results (150). The lungs of hUC-MSCs treated mice showed significantly lower levels of injury-related proteins associated with immunomodulation (C-X3-C motif chemokine ligand 1 (CX3CL1), TNF-α, T cell immunoglobulin and mucin domain (TIM-1), hepassocin, neprilysin), cell survival (osteoprotegerin), and wound healing [MMP-2, leukocyte inhibitory factor (LIF)]. Moreira et al. (150) suggested the intranasal route of delivery as a feasible, non-invasive and effective method that may bear clinical applicability (150).

Elastin expressions stimulated by 90% O_2_ in human lung fibroblasts (HLF) of a hyperoxia-induced rat model of BPD were reduced by intratracheal-delivered UC-MSCs. HLF trans-differentiation into myofibroblasts were also inhibited, indicating that UC-MSCs could inhibit lung elastase activity. These findings showed that UC-MSCs could ameliorate aberrant elastin expression and deposition in the lung of hyperoxia-induced BPD models, possibly through the suppression of TGF-β (151).

### Clinical Translation: From Bench to Bedside

The results following extensive *in vitro* and *in vivo* experiments on animal BPD models using UC-MSCs and hAECs, are promising. This has created great enthusiasm in the scientific community with a surge in clinical trials, offering new hopes of cure for a myriad of diseases including BPD. Notably, hUC-MSCs are among the MSCs that had been extensively investigated over the past decades on various small and larger animal models.

There are currently 11 registered clinical trials of PDSCs and UCDSCs used for BPD listed in the United States National Institute of Health database at https://clinicaltrials.gov, while two are listed in the Australian New Zealand Clinical Trials Registry at https://anzctr.org.au (last accessed on 15 September 2020). At present, clinical trials are also being conducted at various countries including Korea, United States, Spain and China.

These clinical trials have collectively embraced umbilical cord as the main source of MSCs with only a few using hAECs. Most of them are phase 1 clinical trials, focusing on the safety and efficacy of PDSCs and UCDSCs in the treatment and prevention of BPD. Intratracheal and intravenous routes are the two preferred routes of administration, with a wide inter-study variation in the selection of dosage that ranges from 1 million cells/kg body weight up to 30 million cells/kg body weight. A summary of the ongoing and completed clinical trials (excluding 5 follow up studies—NCT03873506, NCT01632475, NCT04003857, NCT01897987, NCT02023788) is in Table 2.

**Table 2:** Ongoing and completed clinical trials on bronchopulmonary dysplasia with placenta and umbilical cord-derived stem cell.

**Identification code**	**Status**	**Cells**	**Phase**	**Study design**			**Age at treatment**	**Target**	**Country**
				**Interventional model**	**Dosage**	**Route of administration**			
NCT04062136	Recruiting	hUC-MSCs	I	Single group	1 × 10^6^/kg (×2)	Intravenous	1–6 months	10	Vietnam
NCT03558334	Recruiting	hUC-MSCs	I	Parallel assignment	1 × 10^6^/kg, 5 × 10^6^/kg	Intravenous	Child, adult, older adult	12	China
NCT03645525	Recruiting	hUC-MSCs	I-II	Parallel assignment	2 × 10^7^/kg	Intratracheal	Up to 3 weeks	180	China
NCT03601416	Not yet Recruiting	hUC-MSCs	II	Parallel assignment	1 × 10^6^/kg, 5 × 10^6^/kg	Intravenous	Up to 1 year	57	China
NCT03631420	Recruiting	hUC-MSCs	I	Single group	3 × 10^6^/kg, 1 × 10^7^/kg, 3 × 10^7^/kg	NA	36–48 weeks	9	Taiwan
NCT04255147	Not yet Recruiting	hUC-MSCs	I	Single group	1 × 10^6^/kg, 3 × 10^6^/kg, 1 × 10^7^/kg	Intravenous	7–21 days	9	Canada
NCT03774537	Recruiting	hUC-MSCs	I-II	Parallel assignment	1 × 10^6^/kg, 5 × 10^6^/kg	Intravenous	3–14 days	20	China
NCT01207869	Unknown	hUC-MSCs	I	Parallel assignment	3 × 10^6^/kg	Intratracheal	Up to 6 months	10	Taiwan
NCT02443961	Recruiting	hUC-MSCs	I	Single group	5 x 10^6^/kg (×3)	Intravenous	1 month to 28 weeks	10	Spain
ACTRN12618000920291	Recruiting	hAECs	I	Other	2 × 10^6^/kg to 3 × 10^7^/kg	Intravenous	14–16 days	24	Australia
^**^ACTRN12614000174684	Completed	hAECs	I	Single group	1 × 10^6^/kg	Intravenous	More than 36 weeks	6	Australia

*HAECs, human amniotic epithelial cells; hUC-MSCs, human umbilical cord mesenchymal stromal/stem cells; hUCB-MSCs, human umbilical cord blood mesenchymal stromal/stem cells; NA, not available*;

** *Published clinical trials*.

hAECs were being explored for their safety and feasibility in treating BPD. Lim et al. (157) conducted the first in-human phase I clinical trial of allogeneic hAECs in 6 preterm babies with BPD via the intravenous route. They reported no immediate adverse events except one of the babies developed transient cardiorespiratory compromise due to pulmonary embolic event. In addition, serum C-reactive protein levels were slightly reduced or remained unchanged 48 h following hAECs administration. One of the six babies died a month after cell administration due to multiorgan failure. The rest of the babies were alive at the time of discharge (median, 174 days of life) (157) and after 2 years of follow up (158) (Table 3).

**Table 3:** Published clinical trials on bronchopulmonary dysplasia with placenta derived stem cells.

**Stem cells**	**Phase**	**Gestational age (weeks)**	**Mean age at treatment (days)**	**Mean birth weight (g)**	**Sample size, n**	**Study designs**		**Results**	**References**
						**Dosage**	**Route**		
Allogeneic hAECs (ACTRN 12614000174684)	I	26	89 days	795	6	1x10^6^/kg	Intravenous	5 alive, 1 died (due to multiorgan failure) 1 transient cardiorespiratory event No other adverse effect	(157)
								During the 2 years follow up: 2 resolved pulmonary hypertension No long-term transplant-related adverse effect	(158)

*HAECs, human amnion epithelial cells*.

### Translational Challenges in Cell-Based Therapy

Although cell-based intervention is hyped as the next therapeutic pillar in medicine, there are still many challenges to be overcome prior to its successful translation into clinical use. Comparing to small molecule and biologic drugs, cell-based therapies are considerably complex and are generally more challenging to control their biological behavior *in vivo*, which has posed a great obstacle to the scientific community toward establishing these cell sources as promising and rewarding therapeutic use in the clinics. In addition, other cell-based therapies such as ECFC and human umbilical vein endothelial cells (HUVEC) that had been comprehensively investigated in adult diseases (159, 160) may be explored for their potential benefits in treating BPD.

Safety remains a primary issue in cell transplantation. The complexity and lack of full understanding on the mode of action of PDSCs and UCDSCs *in vivo* remains a major issue (161). As compared with other small molecule or biologic drugs, these cells are living entities that are capable of metabolism, growth and reaction to environmental stimuli, affecting their therapeutic abilities.

Heterogeneity of PDSCs and UCDSCs may become a barrier that hinders treatment success. Notably, stem cells derived from different anatomic locations of the human placenta, although revealed to share common morphology and immunophenotypic pattern, differ significantly with regards to the numbers of cells in the host tissue, global gene expression patterns and their trilineage differentiation potential *ex vivo*. Careful biomolecular characterization of PDSCs and UCDSCs through genomic, epigenetic, secretomic and proteomic profiling can further refine their overlapping identities and hence reduce heterogeneity.

Standardization in isolation methods is hard to be achieved as different protocols exist for PDSCs/UCDSCs isolation from different anatomic locations. Isolation of cells from human amniotic membrane particularly could be problematic and at risk of cross-contamination due to both AECs and AM-MSCs located close to one another (54). Isolation of hAECs from amniotic fluid may inadvertently harvest trophoblast cells as well (162). Furthermore, the number of cells isolated is often not adequate for clinical use, requiring cell expansion by culture method to achieve a minimum of 1.5-6 × 10^7^ cells per single dose (163). Large-scale production of MSCs is currently made possible with robust controlled bioreactor systems which enable rigorous process monitoring to ensure cell cultivation under optimum controlled conditions (164).

Lastly, it is imperative to note that the risk in transmitting of infections such as viruses, prions and mycoplasma from donors to the immunocompromised premature host although minimal, but extant (165). Stringent safety assurance system with appropriate donor selection and screening as well as employing sensitive screening tests for infectious diseases is mandatory.

## Conclusion and Future Prospects

Currently, there is no single effective therapy for BPD and as such, stem cells have emerged as a potential source of effective therapy. Stem cell insufficiency in preterm infants may be one of the underlying pathological mechanisms for disordered development of alveolar and vascular structures. The placenta and umbilical cord are readily available source of stem cells, although the properties of these stem cells may differ depending on the regions they are isolated from.

To date, there are still very limited studies on the direct comparison between the value of MSCs from different sources, especially between BM-MSCs and P-MSCs/UC-MSCs. It would also be valuable to compare stem cells-derived EVs from various sources such as placenta, bone marrow, and adipose tissue. Accumulating evidence has demonstrated the beneficial effects of these placenta/umbilical cord-derived stem cells on both *in vitro* and *in vivo* experimental animal BPD models. Pre-clinical studies showed they are able to treat established BPD and can prevent BPD from developing in preterm neonates exposed to lung insults. The effects include reduced in the levels of inflammatory mediators such as IL-6 and TNF-α, improved pulmonary angiogenesis, ameliorated lung fibrosis and restored alveolar structures in BPD experimental models. Although paracrine activities seemed to be the most likely mechanism involved, the exact functions of how these stem cells work *in vivo* remain unclear, leaving a knowledge gap in this area.

At the present moment, hAECs and hUC-MSCs are two stem-cell based therapies that are leading the way as potential treatment of BPD. In our opinion and backed by increasing evidence that the “game-changer” lies in the treatment with cell-free products via EVs which appear to yield similar benefits as stem cells. EVs are considered a novel discovery in stem cell biology. Although there are still many uncertainties and questions with regards to this product and that safety of this product has yet to be tested in any phase 1 clinical trial, EVs being cell-free do not have the inherent concerns of uncontrolled transformation. Future studies should investigate the properties of EVs isolated from different regions of placenta and umbilical cord. Lastly, standardized extraction methods and harvesting techniques are necessary to ensure quality and reproducibility of this stem cell product before it is released for use in human translational applications.

## Author Contributions

WKC, YPW, and GCT: writing—original draft preparation. SS, NHAA, and NCK: writing—review. GCT, TYK, and FCC: writing—critical review and editing. YPW, FCC, and GCT: funding acquisition. All authors have read and agreed to the published version of the manuscript.

## Conflict of Interest

The authors declare that the research was conducted in the absence of any commercial or financial relationships that could be construed as a potential conflict of interest.

## Abbreviations

AECs: amniotic epithelial cells
AMSCs: amniotic mesenchymal stromal/stem cells
AM-MSCs: amniotic membrane-derived mesenchymal stromal/stem cells
BM-MSCs: bone marrow-derived mesenchymal stromal/stem cells
BPD: bronchopulmonary dysplasia
ECFCs: endothelial colony-forming cells
ESCs: embryonic stem cells
EVs: extracellular vesicles
hAECs: human amniotic epithelial cells
hUC-MSCs: human umbilical cord mesenchymal stromal/stem cells
L-MSCs: lung-resident mesenchymal stromal/stem cells
LPS: lipopolysaccharide
MSCs: mesenchymal stromal/stem cells
P-MSCs: placental mesenchymal stromal/stem cells
PDSCs: Placenta-derived stem cells
UCDSCs: umbilical cord-derived stem cells
UC-MSCs: umbilical cord mesenchymal stromal/stem cells.
